# The Volumetric Source Function: Looking Inside van der Waals Interactions

**DOI:** 10.1038/s41598-020-64261-4

**Published:** 2020-05-08

**Authors:** Christian Tantardini, Adam A. L. Michalchuk, Artem Samtsevich, Carlo Rota, Alexander G. Kvashnin

**Affiliations:** 1 Skolkovo Institute of Science and Technology, Skolkovo Innovation Center, 3 Nobel Street, Moscow, 121025 Russian Federation; 2 EaStCHEM School of Chemistry and Centre for Science at Extreme Conditions, University of Edinburgh, Edinburgh, United Kingdom EH9 3FD; 3 BAM Federal Institute for Materials Research and Testing, Richard-Willstatter-Str, Berlin, 12489 Germany; 4 CRC Engineering, Via Trieste, 24, 22030 Castelmarte, CO Italy

**Keywords:** Chemistry, Theoretical chemistry, Quantum chemistry

## Abstract

The study of van der Waals interactions plays a central role in the understanding of bonding across a range of biological, chemical and physical phenomena. The presence of van der Waals interactions can be identified through analysis of the reduced density gradient, a fundamental parameter at the core of Density Functional Theory. An extension of Bader’s Quantum Theory of Atoms in Molecules is developed here through combination with the analysis of the reduced density gradient. Through this development, a new quantum chemical topological tool is presented: the volumetric source function. This technique allows insight into the atomic composition of van der Waals interactions, offering the first route towards applying the highly successful source function to these disperse interactions. A new algorithm has been implemented in the open-source code, CRITIC2, and tested on acetone, adipic and maleic acids molecular crystals, each stabilized by van der Waals interactions. This novel technique for studying van der Waals interactions at an atomic level offers unprecedented opportunities in the fundamental study of intermolecular interactions and molecular design for crystal engineering, drug design and bio-macromolecular processes.

## Introduction

Non-covalent interactions (NCIs) are responsible for the dynamics of life. Examples can be drawn from across the physical and life sciences, including the functioning of DNA and proteins, the mechanisms of pharmaceutical action, and the properties of liquid and solid water. To the molecular materials scientist, NCIs are central towards the understanding of both structure and properties, including solubility^1–3^, compressibility^4^ and polymorphism^5^. This includes many technologically important molecular materials such as pharmaceuticals, organic opto-electronics, and energetic materials. Correspondingly, understanding the properties and gaining control of NCIs drives a very active field of research. The term NCI encompasses a broad range of interactions, most notable of which are the hydrogen bond (HB) and the weaker van der Waals (vdW) interactions. While both are examples of NCIs, they differ substantially in their origin^6,7^. On account of their directionality and relative strength, and by no small means their relative conceptual simplicity, the HB is often considered a key design ingredient when developing supra-molecular structures^8–16^. However, HB are less dynamic and less prevalent than vdW interactions^8–16^. The latter are truly ubiquitous and obtaining conceptual control over their use could revolutionize the design and understanding of dynamic systems^17^.

The study of vdW interactions has long been a challenge for scientists working in the field of computational materials science. To capture these interactions, most modern techniques require the inclusion of empirical or semi-empirical corrections to the quantum mechanical energies^18–23^. This has rendered the thorough study of vdW interactions very difficult. Recent years have seen the development of energy decomposition methods, such as PIXEL^24^ and symmetry-adapted perturbation theory (SAPT)^25^. Based on decomposition of the electron density (i.e., PIXEL) or of the wavefunction (i.e., SAPT), these methods offer numerical calculation of dispersive interactions. The structural interpretation of these methods can be difficult, particularly where no well-defined bond path is present. This is the case for vdW interactions and represents a substantial barrier to harnessing these interactions for design applications.

Recently, Johnson *et al*.^26–29^ developed an approach to visualize NCIs based on topological analysis of the electron density, dubbed NCI surfaces. Within their method, the reduced density gradient (RDG) is analyzed and an interaction map is generated where $s({\bf{r}})\to 0$; *i.e*. critical points. The magnitude of ${\rm{\rho }}({\bf{r}})$ is used to scale the relative strengths of these interactions, and the nature of the interaction is identified by analysis of the Hessian of the Laplacian of electron density. This analysis leads to a highly visual representation of NCIs, without need for expert knowledge in electron density analysis. The intuitive nature of this approach has made it immensely popular among both the experimental and theoretical communities. In particular, NCI surface analysis has found widespread applications in the materials, chemistry, and biology communities, offering new insight into the structure of bonding interactions^26–29^ and self-assembly phenomena^10,29–31^. Furthermore, this technique has allowed for the identification of non-conventional hydrogen bonds, which are typically unobserved within the frameworks of other theoretical approaches^31–35^.

Despite its conceptual power, analysis of NCI surfaces does not permit atomic-level interpretation. That is to say that while the NCI surface shows the entire interaction surface between molecules, no information is obtained as to which atoms contribute to this interaction. Such information would offer invaluable insights into the tunability of NCIs. A topological tool – the source function (SF)^36–45^ – is already known to provide this insight, doing so within the framework of the Quantum Theory of Atoms in Molecules (QTAIM)^43^. The NCIs are typically descripted by SF at well-defined critical points. Albeit, the SF reconstruction along a line, on a surface or within a volume were previously introduced and discussed, both for the electron density and the electron spin density^46–48^. Here, we describe an interesting application of the SF double integration procedure through its combination with the study of NCI surfaces. This extension allows, for the first time, insight into the atomic contributions to NCIs, and hence represents a new tool in the targeted design of molecular recognition. Through this work, we develop a fundamental extension to the traditional bond critical point^43^ (BCP) seen by QTAIM, dubbed the vdW volume (${V}_{vdW}$). Extension of the SF to accommodate the ${V}_{vdW}$ leads to generation of a further development: the volumetric source function (VSF). Analysis of NCI surfaces by the VSF provides, for the first time, a chemically intuitive, quantitative approach to the study of non-directional interactions. This technique adds a new method to the chemists’ toolbox to investigate the nature and structure of vdW interactions, with unique atomic-level insight.

To demonstrate the potential of our extension to QTAIM a brief introduction to the fundamental theories and developments is provided, which is followed by the description of the corresponding algorithmic developments. The development of theoretical considerations is tested on acetone, adipic, and maleic acids molecular crystals discussing the interpretation of vdW interactions with the new topological tool so called VSF here presented.

## Theoretical Background

### Introduction to QTAIM and the source function

QTAIM is based on the separation of molecules into distinct atomic subunits, familiar to chemists. These atomic subunits (known as atomic basins) are delimited by surface so called zero flux surface. Such surface is made by an infinity of points ${\bf{r}}$, for which the dot product of the gradient of electron density, $\nabla {\rm{\rho }}$, and the normal vector, $\hat{{\bf{n}}}$, is zero (zero-flux boundary condition).

1 $$\nabla {\rm{\rho }}({\bf{r}})\cdot \hat{{\bf{n}}}({\bf{r}})=0$$

This is to say that the surface of an atom is defined by the set of vectors $(\hat{{\bf{n}}})$ that align perpendicular to these points of $\nabla {\rm{\rho }}=0$. All points which sit on this surface are denoted ${{\bf{r}}}_{s}$. Thus, there is no change (or flux) of electron density across this surface, and it is ‘zero-flux’. Particular critical points (namely where ${\nabla }^{2}{\rm{\rho }}({\bf{r}})$ is negative in only one direction) are known as BCPs and define the unique bond path of covalent (or semi-covalent, e.g. HB) interactions. In order to describe bonding at BCPs intuitively, an extension to QTAIM was developed, dubbed the SF. The SF describes the contribution of each atomic basin to the BCP and hence describes the atomic contributions to individual covalent bonds^43^, or intermolecular hydrogen bonds^44,45^. Only in very specific cases the SF can be used with success to describe bonding in vdW interactions^45^, because in principle vdW interactions may be described in terms of SF reconstructions as for any other point or surface or volume, but there might arise serious numerical accuracy problems. The electron density at point ${\bf{r}}$ that comes from atomic basin Ω is given by the sum of two contributions: (i) the integral of ${\nabla }^{2}{\rm{\rho }}({\bf{r}}{\boldsymbol{\text{'}}})$ evaluated over all points ${{\bf{r}}}^{\text{'}}$ within $\Omega$, each weighted as an inverse function of its distance from the point of interest ${|{\bf{r}}-{\bf{r}}\text{'}|}^{-1}$; and (ii) the flux of the electric field density, ${\rm{\varepsilon }}({\bf{r}}-{{\bf{r}}}_{s})$, across the boundary of Ω, calculated at ${\bf{r}}$. This term depends on the electron density of the surface of Ω, ${\rm{\rho }}({{\bf{r}}}_{S})$^36–45^. The electron density at point ${\bf{r}}$ within an atomic basin Ω can be written as,

2 $$\rho ({\bf{r}})=-\frac{1}{4\pi }\left\{{\int }_{\varOmega }d{{\bf{r}}}^{\text{'}}\frac{{{\rm{\nabla }}}^{2}\rho ({{\boldsymbol{r}}}^{\text{'}})}{|{\bf{r}}-{{\bf{r}}}^{\text{'}}|}+{\oint }_{{S}_{\varOmega }}dS({{\bf{r}}}_{S})\cdot \varepsilon ({\bf{r}}-{{\bf{r}}}^{\text{'}})\right\}$$

For a closed system with boundary at infinity the second term of Equation 2 is reduced to zero. For a system with more than one atomic basin, ${\rm{\rho }}({\bf{r}})$ becomes a sum of the contribution of each atomic basin, with each individual contribution known as the SF for the respective basin,

3 $$\rho ({\bf{r}})=-\frac{1}{4\pi }{\int }_{\varOmega }d{{\bf{r}}}^{\text{'}}\frac{{\nabla }^{2}\rho ({{\bf{r}}}^{\text{'}})}{|{\bf{r}}-{{\bf{r}}}^{\text{'}}|}=-\frac{1}{4\pi }\sum _{\varOmega }{\int }_{\varOmega }d{{\bf{r}}}^{\text{'}}{\rm{LS}}({\bf{r}},{{\bf{r}}}^{\text{'}})\equiv \sum _{\varOmega }{\rm{SF}}({\bf{r}},\varOmega )$$

The integrand in Equation 3 is defined as Local Source (LS).

4 $${\rm{LS}}({\bf{r}},{{\bf{r}}}^{\text{'}})\propto \,-{\nabla }^{2}\rho ({{\bf{r}}}^{\text{'}})$$

This definition is useful as it provides characterization of the local contributions to point ${\bf{r}}$*.* When ${\nabla }^{2}\rho ({\bf{r}}\text{'}) > 0$, then $LS({\bf{r}},{\bf{r}}\text{'}) < 0$ and ${\bf{r}}\text{'}$ is a *“sink”* (depleting the electron density); when ${\nabla }^{2}\rho ({\bf{r}}\text{'}) < 0$, then $L{\rm{S}}({\bf{r}},{\bf{r}}\text{'}) > 0$ and ${\bf{r}}\text{'}$ is a *“source”* (enriching the electron density).

### Non-covalent interaction surfaces

The RDG^26–29^ is a fundamental quantity defined within DFT formalism. Derived from the electron density at point ${\bf{r}}$, ${\rm{\rho }}({\bf{r}})$, and its first derivative, $\nabla {\rm{\rho }}({\bf{r}})$, the RDG is defined as,

5 $$s({\bf{r}})=\frac{|\nabla {\rm{\rho }}({\bf{r}})|}{2{(3{{\rm{\pi }}}^{2})}^{1/3}{\rm{\rho }}{({\bf{r}})}^{4/3}}$$

For regions far from the various nuclei of a system (i.e. where density decays exponentially to zero), the RDG adopts large positive values. In contrast, the RDG values approach zero for regions of covalent and non-covalent bonding. Hence, the magnitude of *s* offers a good indication of the position of NCIs. The nature of the NCI is subsequently defined by analyzing the Laplacian of electron density ${\nabla }^{2}{\rm{\rho }}({\bf{r}})$. To characterize interactions, the Laplacian is often decomposed into its three principal axes of maximum variation, the three eigenvalues ${{\rm{\lambda }}}_{{\rm{n}}}$ of the Hessian of electron density, ${\nabla }^{2}{\rm{\rho }}({\bf{r}})={{\rm{\lambda }}}_{1}+{{\rm{\lambda }}}_{2}+{{\rm{\lambda }}}_{3}({{\rm{\lambda }}}_{1}\le {{\rm{\lambda }}}_{2}\le {{\rm{\lambda }}}_{3})$. As per convention, ${{\rm{\lambda }}}_{1}$, ${\lambda }_{2}$ and ${{\rm{\lambda }}}_{3}$ are in ascending order. Then for BCPs it is clear that ${{\rm{\lambda }}}_{3}$ is always greater than zero. This was arbitrarily assumed, in view of the fact that the low RDG isosurfaces bound volumes where in most cases RDG goes to zero and so enclose an electron density critical point (CP). When this is a BCP (bonding interaction) ${\lambda }_{2}$ is negative and when this CP is a ring CP (associated to non-bonding/repulsive interactions at the ring CP between the atoms forming the ring) ${\lambda }_{2}$ is positive and for vdW interactions ${{\rm{\lambda }}}_{2}\approx 0$. Hence the nature of interactions at points of ${\rm{s}}({\bf{r}})\to 0$ can be defined by the corresponding value of ${\rm{\rho }}({\bf{r}})\cdot {\rm{sign}}({{\rm{\lambda }}}_{2})$. It is generally accepted that the magnitude of ${\rm{\rho }}({\bf{r}})$ corresponds to the relative strength of interaction. As a rule of thumb, it has been suggested that the limits $\pm 0.02$ a.u. is used to distinguish between bonding regimes, with bonding interactions found at ${\rm{\rho }}({\bf{r}})\cdot {\rm{sign}}({{\rm{\lambda }}}_{2}) < -0.02$ a.u., vdW interaction in regions where $-0.02 < {\rm{\rho }}({\bf{r}})\cdot {\rm{sign}}({{\rm{\lambda }}}_{2}) < 0.02$ a.u., and steric repulsion in region with ${\rm{\rho }}({\bf{r}})\cdot {\rm{sign}}({{\rm{\lambda }}}_{2}) > \,0.02$ a.u^26–29^.

## Development of volumetric source function

The above discussion of SF relates to the decomposition of atomic basin contributions to a well-defined point. This is generally taken to be a BCP, and hence SF has traditionally been used to interpret covalent and well-defined NCIs such as HBs. Unfortunately, due to their non-directionality, vdW interactions are not associated with such well-defined critical points. Hence, these interactions have remained beyond the scope of QTAIM analysis. In order to extend QTAIM for this purpose, we define a ${V}_{vdW}$ via the infinite points within the volume defined by NCI analysis, ${{\bf{r}}}_{vdW}\,\in {V}_{vdW}$. The number of electrons within ${V}_{vdW}$ for a closed system with boundary at infinity is given by the sum on all $M$ atomic basins of VSF. Such equation is defined as the integral of SF on the ${V}_{vdW}$ giving the contribution of an atomic basin to such volume,

6 $${n}_{{V}_{vdW}}^{e}=\mathop{\sum }\limits_{{\boldsymbol{\Omega }}=1}^{M}{\rm{VSF}}({V}_{vdW},\varOmega )=\mathop{\sum }\limits_{{\boldsymbol{\Omega }}=1}^{M}{\int }_{{V}_{vdW}}{\rm{SF}}({\bf{r}},\varOmega ){d}^{3}{\rm{r}}$$

## Method

**Scheme 1:**
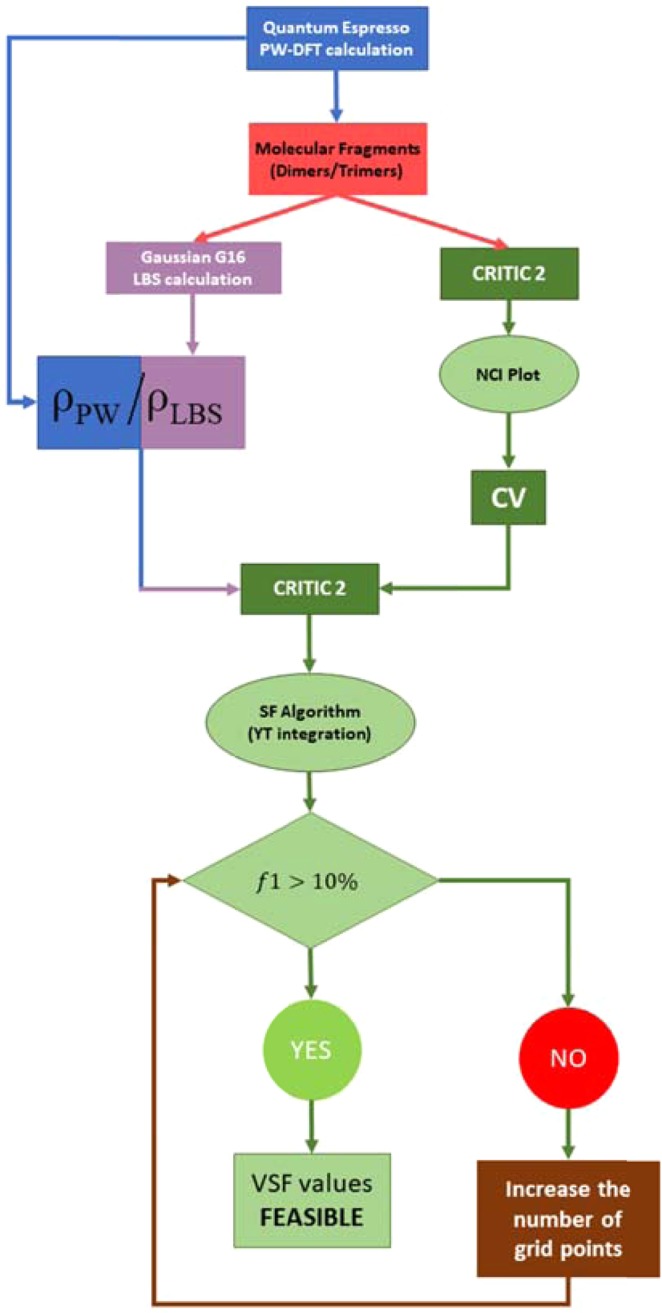
Workflow diagram describing the methodology used for correct reproduction of the VSF using localised basis sets (LBS) within the MP2/aug-cc-pVTZ and plane waves (PW) electron densities. The function *f*1 is the Gatti’s reliability parameter^42^, which describes the percentage error in the reconstruction of the electronic density within the vdW volume (${V}_{vdW}$).

The general workflow developed for calculation of the VSF is summarized in Scheme 1. In order to investigate the nature of vdW interactions within the solid state, the input structures were first relaxed in the solid-state using plane-wave Density Functional Theory (PW-DFT). The electron density of the relaxed structure was subsequently obtained with a plane wave kinetic energy cut-off of 100 Ry. This value has been previously shown to facilitate the reliable calculation of the SF^48^.

The resulting electron density was analyzed for NCI surfaces using the NCI*plot* code, as implemented in CRITIC2^49,50^. The NCI surfaces were generated for $-0.02\,\le \rho \cdot {\rm{sign}}({{\rm{\lambda }}}_{2})\le 0.02$ a.u. The NCI surfaces corresponding to vdW dimers that compose the reticular motif of acetone, adipic and maleic acids crystal structures were subsequently isolated and taken as definition of the ... To assess the suitability of PW basis sets, the electron density in the dimers was recalculated with localized basis set (LBS) at the MP2^51–55^/aug-cc-pVTZ^56^ level. This density was subsequently projected onto the NCI surface obtained above (*i.e*. onto the ${V}_{vdW}$). The SF for each atomic basin on each sampled point within ${V}_{vdW}$ was calculated using YT integration scheme^57^. Only the points characterized by 10^-3^ <ρ <10^-6^ *e*/bohr3 were sampled within ${V}_{vdW}$ to calculate the number of expected electrons inside it. The expected number of electrons, ${n}_{{V}_{vdW}}^{e}$, within ${V}_{vdW}$ from each atomic basin, ${\rm{VSF}}({V}_{vdW},\varOmega )$, was numerically approximated in according to Monte Carlo method^58^ by the arithmetic mean of ${\rm{SF}}({{\bf{r}}}_{{\boldsymbol{i}}},\varOmega )$, as in,

7 $${\rm{VSF}}({{\rm{V}}}_{{\rm{vdW}}},\varOmega )\approx {V}_{vdW}\cdot \frac{1}{{\rm{N}}}\mathop{\sum }\limits_{i=1}^{{\rm{N}}}{\rm{SF}}({{\bf{r}}}_{{\boldsymbol{i}}},\varOmega )$$

With 1,…,N the independent realization of random variable ${\bf{r}}$ within ${V}_{vdW}$.

A sampling of the number of points needed to get a stable value of number of electrons within ${V}_{vdW}$ using Monte Carlo method^58^ was performed with Metropolis-Hastings approach^59^. This was done to evaluate the error of RDG estimation for ${V}_{vdW}$ due to the number of points used for numerical integration. The error estimation for RDG was possible due to non-divergent values of $\nabla {\rm{\rho }}$ and ${{\rm{\rho }}}^{4/3}$,

8 $${\rm{RDG}} \sim \frac{\nabla \rho }{{\rho }^{4/3}};\,\nabla \rho \to \frac{\delta \rho }{\rho };\,{\rho }^{4/3}\to \frac{4}{3}\rho $$

9 $${\left(\frac{{\rm{RDG}}}{RDG}\right)}^{2}={\left(\frac{\varDelta \nabla {\rm{\rho }}}{\rho }\right)}^{2}+{\left(\frac{4}{3}\frac{{\rm{\delta }}{\rm{\rho }}}{\rho }\right)}^{2}={\left(\frac{{\rm{\delta }}{\rm{\rho }}}{\rho }\right)}^{2}+c{\left(\frac{{\rm{\delta }}{\rm{\rho }}}{\rho }\right)}^{2}$$

The error was found to achieve the asymptote for 1000 points. Thus, to guarantee the most feasible NCI description ${V}_{vdW}$ were sampled with more than 1000 points neglecting the point with ρ less than 10^-6^ *e*/bohr3.

## Computational Details

Initial structures for each of the molecular crystals used in this study were taken from the Cambridge Crystallographic Data Centre: acetone, ref. code HIXHIF05;^60^ adipic acid, ref. code ADIPAC12;^61^ maleic acid, ref. code MALIAC14^62^. These structures were fully relaxed using PW-DFT, as implemented in Quantum ESPRESSO (v6.4)^63^. The PW86PBE^64,65^ exchange-correlation functional was used in combination with the exchange-hole dipole moment (XDM) dispersion correction with damping factors a_1_=0.6836 and a_2_=1.5045^66–68^. XDM uses the interaction of induced dipoles (and higher-order multipoles) to model dispersion: the source of the instantaneous dipole moments is taken to be the dipole moment of the exchange hole^66–68^. The wavefunction was expanded in a plane wave basis set to 100 Ry, and Brillouin zone integration was performed on 6×6×6 Monkhorst-Pack *k*-point grid^69^. The SCF convergence was accepted when less than 10^-8^ Ry, and geometry relaxation accepted once residual atomic forces fell below 10^-8^ Ry/bohr. Comparison of the experimental and relaxed structures are given in Table E.S.I. 1. The NCI surfaces were generated from the PW density, using the NCI implementation within CRITIC2. Values of $0.45\le s\le 0.55$ for computational generated densities as suggested in the original NCI work^26–29^ were chosen. The VSF was calculated using an in-house code, according to the work scheme shown in Scheme 1. The LBS electron density used in the final integration step was obtained for the isolated dimers using Gaussian v.16^70^ at the MP2^51–55^ level with aug-cc-pVTZ basis set^56^ for each atom. The dimers extracted for electron density analysis were used as input for SAPT^25^ analysis of NCI energies. The scaled SAPT zero energies were calculated within the PSI4 software using the jun-cc-pVDZ basis set^71^, the bronze level SAPT method identified by Parker *et al*. with overall error of $\pm$ 2.05 kJ/mol^72^.

To offer a more direct numerical comparison the VSF values in this article they are provided in term of VSF%,

10 $${\rm{V}}{\rm{S}}{\rm{F}}({V}_{vdW},\varOmega ){\rm{ \% }}=\frac{{\rm{V}}{\rm{S}}{\rm{F}}({V}_{vdW},\varOmega )}{{n}_{{V}_{vdW}}^{e}}\cdot 100$$

## Results and Discussion

**Figure 1:**
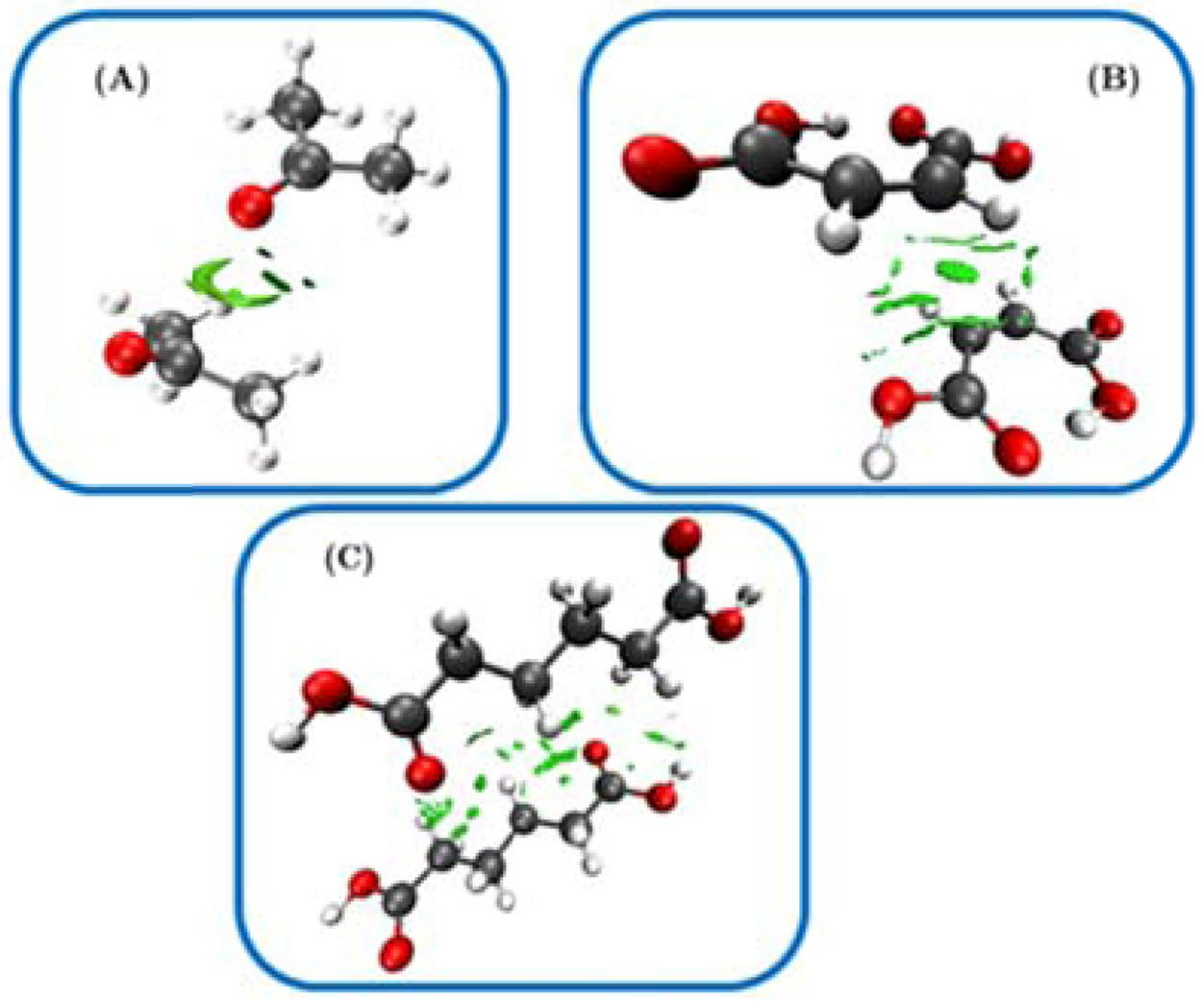
NCI surfaces ($s=0.5$) for vdW-bound dimers extracted from relaxed solid-state structures. Dimers correspond to (**A**) acetone, (**B**) maleic acid and (**C**) adipic acid. Carbon atoms are grey, oxygen – red, hydrogen – white.

The NCI surfaces representing vdW interactions with $\,{\rm{\rho }}\cdot {\rm{sign}}({{\rm{\lambda }}}_{2})\approx 0$ a.u. (i.e., from -0.02 to + 0.02 a.u.)^26–29^ for the dimers within acetone, adipic and maleic acids crystal structures were calculated on PW density and isolated (Fig. 1). Hence, each of the selected cases represents a vdW dimer under periodic condition^26–29^.

As analogue to the role of ${{\rm{\rho }}}_{bcp}$ in HB systems^44,45^, one would expect that the value of ${n}_{{V}_{vdW}}^{e}$ should correlate to the total interaction energy across the dimer. As previously explored by Saleh *et al*.^73^ (2015) the correlation between the value of electron density and the interaction energies calculated with SAPT^25^ interaction energies, Table 1 and once again we do not find any notable correlation. This suggests that – unlike ${{\rm{\rho }}}_{bcp}$ – the integral of electron density has different character and is not directly indicative of the strength of this category of intermolecular interaction. This is presumably due to the fact that a ${V}_{vdW}$ composed of points with ${{\rm{\lambda }}}_{2}\approx 0$ contains loci of both attractive and repulsive character.

**Table 1:** Number of electrons, ${n}_{{V}_{vdW}}^{e}$, within vdW volumes (${V}_{vdW}$) coming from local basis set electron density for each of the three vdW dimers: acetone (AC), adipic acid (AA) and maleic acid (MA).

	AC	AA	MA
${{\boldsymbol{n}}}_{{{\boldsymbol{V}}}_{{\boldsymbol{vdW}}}}^{{\boldsymbol{e}}}$	0.1692	0.0566	0.0333
**SAPT Total**	−5.697	−25.417	−29.080
**Ele**.	−5.008	−18.686	−17.526
**Exch**.	13.309	16.331	25.063
**Ind**.	−2.833	−4.306	−6.360
**Disp**.	−11.166	−18.757	−22.305

Note that symmetry-adapted perturbation theory (SAPT) energies are given in kJ/mol. The SAPT energy is decomposed into electrostatic (ele.), exchange (exch.), inductive (ind.) and dispersion (disp.) contributions.

### VSF from localized basis set (LBS) electron density

In order to consider the validity of the VSF, we adopt the approach suggested by Gavezzotti for electron density decomposition^24^. Here, the nuclear positions are extracted from the relaxed crystal structure and the electron density is recalculated using a LBS with the post Hartree-Fock correlation scheme, MP2. This approximation is supported by the fact that the Laplacian of electron density exponentially decreases far away from the nucleus as ${e}^{-{x}^{2}}$, where *x* represents the relative distance from nucleus at the point **R**^43^. The dimers can hence be extracted from crystals given that the primary neighbours will negligibly affect the intermolecular interactions between dimer pairs. To best correlate our values to crystalline structures, the electron density obtained using the MP2/LBS approach were projected onto the ${V}_{vdW}\,$s calculated from periodic calculations. Decomposition of LBS-based ${{\rm{\rho }}}_{{V}_{vdW}}$ *via* Equation 6 yields mixed results for the reconstruction of the

**Figure 2:**
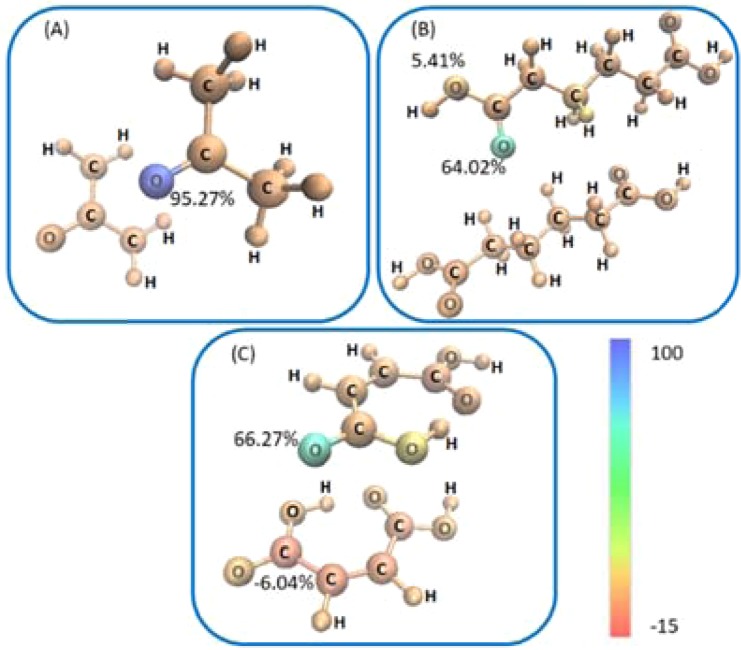
Average atomistic local basis set volumetric source function (VSF) values for (**A**) acetone, (**B**) adipic and (**C**) maleic dimers, based on ${n}_{{V}_{vdW}}^{e}$. Atoms are coloured according to their respective VSF% contributions to the ${V}_{vdW}$ shown in Fig. 1. The colour scale is given. As indication, atom VSF values are given to highlight largest values in each case. A full list of VSF is given in Tables E.S.I. 2–4.

${n}_{{V}_{vdW}}^{e}$, Table 1. According to Gatti’s reliability parameter^42^,

11 $$f1=\frac{{n}_{{V}_{vdW}}^{e}-{\sum }_{\Omega }{\rm{VSF}}({V}_{vdW},\Omega )}{{n}_{{V}_{vdW}}^{e}}\cdot 100$$

we obtain values $f1$ of 0.14%, 1.19% and 1.32% for acetone, adipic and maleic acids, respectively. Considering the VSF more closely, we simply decomposed ${n}_{{V}_{vdW}}^{e}$ into its atomic contributions for each dimer starting from the electron density contribution of each atom inside the ${V}_{vdW}$, Fig. 2. In each structure there is a single oxygen atom which clearly dominates in its contribution to ${n}_{{V}_{vdW}}^{e}$. Hence, there is a single oxygen atom in each case which can be suggested as being largely responsible for the vdW interaction. There is no corresponding density sink visible in any of these three systems. Instead, it appears that the electron density donated by the oxygen atom is spread largely uniformly across the remaining atoms in the system. This is consistent with prevailing theories of vdW interactions, suggesting that the interaction is indeed dependent on the total number of atoms into which the donated density can distribute.

### VSF from plane waves (PW) electron density

Recent work has, however, demonstrated that the SF could be reliably reproduced from PW densities, provided sufficiently high kinetic energy cut-offs^48^. It is not immediately evident whether the same holds for reconstruction of regions with very low electron density, as ${V}_{vdW}$, is possible and if from SF evaluated in such regions is possible to calculate VSF. We therefore extend the above discussion to consideration of VSF as obtained by a PW basis, with contributions to the ${V}_{vdW}$ taken from atoms throughout the entire structure, *i.e*. throughout the unit cell. Hence, the ${n}_{{V}_{vdW}}^{e}$ will be different with respect to that calculated from LBS electron density of extracted dimers. As the case for LBS electron densities, the $f1$ values for PW electron densities were obtained for acetone, adipic and maleic acids (i.e., 22.18%, 26.33% and 30.22% respectively). As expected from the above discussion, $f1$ values for PW electron densities are highest than for LBS electron densities.

**Figure 3:**
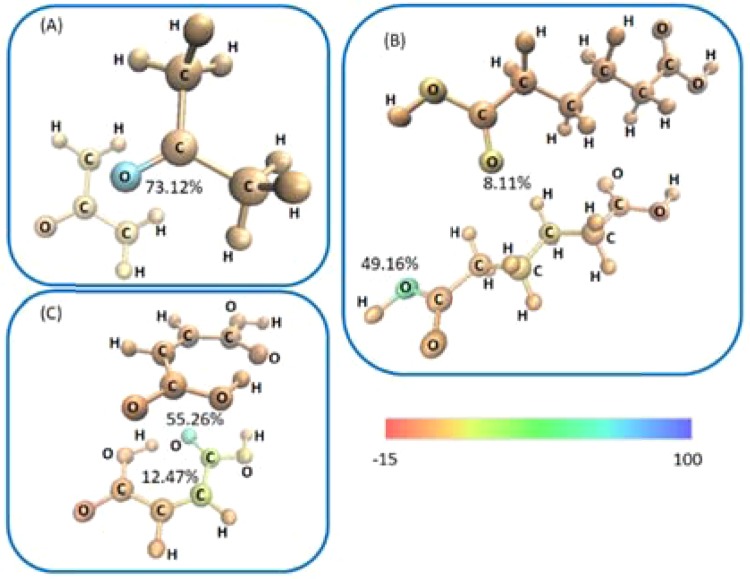
Average atomistic plane waves volumetric source function (VSF) values for (A) acetone, (B) adipic and (C) maleic acids, based on ${n}_{{V}_{vdW}}^{e}$. Atoms are coloured according to their respective VSF% contributions to the critical volume shown in Fig. 1. The colour scale is given. As indication, atom VSF values are given to highlight largest values in each case. A full list of VSF is given in Tables E.S.I. 5–7.

As compared with the atomistic VSF contributions obtained by LBS above, the qualitative picture obtained for acetone is well reproduced, Fig. 3A. Again, only a single oxygen atom contributes to the NCI surface, while the remaining atoms exhibit approximately equal acceptor contributions. Unfortunately, the systems which exhibit much lower values of electron density and consequent ${n}_{{V}_{vdW}}^{e}$ is poorly reproduced when the VSF is generated by PW densities, Fig. 3B,C. Hence, the attempt to use PW densities to derive SF values at the ${V}_{vdW}$ was seen to not be so feasible like the one with LBS densities. Albeit in previously works SF values derived by PW densities were seen comparable with those obtained with LBS densities at the BCP^43^. This can be due to the fact that the reconstruction of electron density into a point is amenable of the value that it itself assumes at that point. Thus, for electron densities in the order of 10^-3^ as seen at the BCPs PW densities work well, but for electron densities less than 10^-3^ *e*/bohr^3^ PW densities are not enough even if they are taken in large numbers.

Furthermore, a general note it is worth mentioning that the atomistic VSF contributions, Tables E.S.I. 5–7, suggests that atoms which exists within unit cell but outside of the dimer pairs contribute on average no more than 1% to ${n}_{{V}_{vdW}}^{e}$. Thus, it is clear that all atoms within the periodic structure *do* contribute to the structure of the ${V}_{vdW}$, but the atomic contribution for those are not directly involved is reasonable neglectable confirming ulterior the work of Gavezzotti with PIXEL^24^.

## Conclusions

Here we outline a different approach to deconvolve vdW interactions in terms of their atomic composition, which description has been a long-standing challenge for theorists. This approach is rooted in the QTAIM^56^ and represents a vdW analogue to the previously developed SF analysis. This development includes the description of a ${V}_{vdW}$ and the corresponding VSF. Based on the approach for electron density decomposition proposed by Gavezotti^24^, it was found that electron densities based on the localized basis set allowed reliable calculation of VSF. Analysis in this way suggests that vdW surfaces are generated through the charge donation of a single heteroatom, here oxygen. The reciprocating charge accepting behaviour is shared across the remaining atoms, consistent with prevailing theories of vdW interactions. This suggests that the VSF method indeed presents a consistent picture of these weak interactions. Despite correctly reproducing the number of electrons, ${n}_{{V}_{vdW}}^{e}$, inside the ${V}_{vdW}$, plane wave basis sets were found less capable of providing reliable calculation of VSF. This was particularly true for systems with low values of electron density within the ${V}_{vdW}$, and is presumably due to the inaccuracies associated with reconstructing points of low electron density. However, this work reports on a new approach for the quantum chemical topological investigation of weak vdW interactions. The methodology is based on empirically definition of vdW interaction using a fundamental dimensionless quantity in DFT that describes the deviation from a homogeneous electron distribution so called RDG^73–75^. This leads to atomistic-level detail of the interacting surface and hence offers the first approach for rational design of these ubiquitous interactions.

## Supplementary information

Supplementary Information.
