# Supplementary material for: The Volumetric Source Function: Looking Inside van der Waals Interactions
Source: Sci Rep. 2020 May 8;10:7816. doi: 10.1038/s41598-020-64261-4 (PMC7210285; doi:10.1038/s41598-020-64261-4)
Supplement: Supplementary file 1 — Supplementary Information. [file 41598_2020_64261_MOESM1_ESM.pdf]

Electronic Supporting Information

The Volumetric Source Function: Looking Inside van der Waals Interactions‡

Christian Tantardini[1], Adam A. L. Michalchuk[2,3], Artem Samtsevich[1] Carlo Rota[4] and Alexander G. Kvashnin[1]

[1]Skolkovo Institute of Science and Technology, Skolkovo Innovation Center, 3 Nobel Street, Moscow, Russian Federation, 121025.

[2]EaStCHEM School of Chemistry and Centre for Science at Extreme Conditions, University of Edinburgh, Edinburgh, United Kingdom, EH9 3FD.

[3]BAM Federal Institute for Materials Research and Testing, Richard-Willstatter-Str, Berlin, Germany, 12489.

[4] Polytechnic University of Milan, Piazza Leonardo da Vinci, 32, 20133 Milano MI, Italy.

‡Dedicated at the carrier of Dr. Carlo Gatti.

*Correspond to Christian Tantardini (christiantantardini@ymail.com)

E.S.I. 1. Table of full **r**elaxed Molecular Crystals*

	a	***b***	***c***	**α**	**β**	**γ**	**V**	***dV***
		**(Å)**			**(Degree)**		**(Å^3)**	
**Acetone^{exp}**	6.3925(17)	5.3424(14)	10.733(3)	90	90	90	336.546	
**Acetone^{calc}**	6.19328	5.00587	10.66951	90.00	90.00	90.00	330.784	-5.762
**Adipic^{exp}**	6.7666(5)	6.9992(5)	7.7180(5)	93.794(4)	104.321(4)	102.689(4)	342.703	
**Adipic^{calc}**	6.76874	6.60612	8.05923	93.7350	104.6638	102.1951	338.073	-4.630
**Maleic^{exp}**	7.1511(8)	10.1107(11)	7.6405(10)	90	119.405(8)	90	481.260	
**Maleic^{calc}**	6.90595	10.18462	7.52825	90.0008	118.3630	89.9964	465.932	-15.328

*The measurements were given with the accuracy reported within files *.cif deposed in the CSD data bank.

E.S.I 2. Table of volumetric source function (VSF) values for acetone dimer calculated with LBS.

Atomic Coordinates / (Å)

atoms	X	Y	Z	VSF	VSF%
1 O	1.872332	0.871424	2.664153	1.618E+00	95.27
2 C	1.609845	2.074688	2.664156	1.662E-02	0.98
3 C	1.401641	2.838489	1.381186	4.880E-03	0.29
4 C	1.401641	2.838489	3.947121	7.779E-03	0.46
5 H	1.824607	2.297581	0.530262	-3.361E-03	-0.20
6 H	1.824607	2.297581	4.798044	4.331E-03	0.26
7 H	0.317404	2.946427	1.2275	1.100E-02	0.65
8 H	0.317404	2.946427	4.100806	8.064E-03	0.47
9 H	1.819038	3.848058	1.448504	1.299E-02	0.77
10 H	1.819038	3.848058	3.879802	9.389E-03	0.55
11 C	4.830339	0.22993	3.947166	-8.067E-04	-0.05
12 C	4.830339	0.22993	1.38114	-9.407E-03	-0.55
13 C	4.62199	-0.53382	2.664184	-1.500E-02	-0.88
14 H	4.412966	1.239515	3.87993	-7.616E-04	-0.04
15 H	4.412966	1.239515	1.448377	-4.449E-04	-0.03
16 H	5.914591	0.337834	4.100771	9.540E-03	0.56
17 H	5.914591	0.337834	1.227536	1.307E-02	0.77
18 H	4.407425	-0.311	4.798094	5.033E-03	0.30
19 H	4.407425	-0.311	0.530212	-5.114E-04	-0.03
20 O	4.359503	-1.73709	2.664182	5.614E-03	0.33
Tot	[redacted]			1.696E+00	99.86

E.S.I. 3. Table of volumetric source function (VSF) values for adipic dimer calculated with LBS.

	Atomic Coordinates / (Å)				
atoms	X	Y	Z	VSF	VSF%
1 O	-0.11192	0.466565	1.636697	7.995E-01	64.02
2 C	-0.77927	1.365859	1.105792	9.756E-04	0.08
3 O	-0.96838	1.454951	-0.2087	6.750E-02	5.41
4 C	-1.4808	2.454878	1.870327	6.788E-03	0.54
5 H	-0.52379	0.681377	-0.70646	9.847E-03	0.79
6 H	-2.55078	2.36612	1.630808	2.394E-02	1.92
7 C	-1.2698	2.438124	3.380522	4.861E-02	3.89
8 H	-1.16278	3.412801	1.434948	3.247E-02	2.60
9 H	-1.47934	1.432809	3.773898	1.443E-01	11.56
10 C	-2.16881	3.464172	4.081334	4.575E-03	0.37
11 H	-0.21438	2.644092	3.606197	3.977E-02	3.18
12 H	-3.22423	3.258204	3.855659	1.488E-02	1.19
13 H	-1.95927	4.469487	3.687958	2.359E-02	1.89
14 C	-1.95781	3.447418	5.591529	-9.884E-03	-0.79
15 H	-2.27583	2.489495	6.026908	-8.881E-03	-0.71
16 C	-2.65934	4.536437	6.356063	-2.175E-03	-0.17
17 H	-0.88783	3.536176	5.831047	1.062E-02	0.85
18 O	-3.32669	5.435731	5.825158	1.500E-02	1.20
19 O	-2.47024	4.447345	7.670556	7.902E-03	0.63
20 H	-2.91483	5.220919	8.168315	5.351E-03	0.43
21 O	1.415554	2.223022	5.955664	1.434E-02	1.15
22 C	1.039295	1.063606	6.178543	-1.562E-02	-1.25
23 C	1.27303	-0.09403	5.248988	-1.641E-03	-0.13
24 O	0.352701	0.714249	7.268946	7.103E-03	0.57
25 H	1.567348	-0.95017	5.871647	1.923E-02	1.54
26 H	0.289402	-0.35048	4.824995	-3.751E-02	-3.00
27 C	2.282598	0.144907	4.132495	-3.684E-03	-0.29
28 H	0.192238	1.516537	7.877827	7.245E-03	0.58
29 C	2.50865	-1.13071	3.313649	-1.602E-03	-0.13
30 H	1.926936	0.951941	3.478002	-1.014E-02	-0.81
31 H	3.235017	0.479812	4.563875	1.813E-02	1.45

32 H	1.556231	-1.46561	2.882269	-1.839E-02	-1.47
33 H	2.864311	-1.93774	3.968142	1.350E-02	1.08
34 C	3.518218	-0.89177	2.197155	-8.376E-04	-0.07
35 C	3.751953	-2.04941	1.2676	-4.420E-03	-0.35
36 H	4.501847	-0.63532	2.621149	8.600E-03	0.69
37 H	3.2239	-0.03563	1.574497	-3.234E-03	-0.26
38 O	4.438546	-1.70005	0.177198	1.935E-03	0.15
39 O	3.375694	-3.20882	1.49048	2.670E-03	0.21
40 H	4.599009	-2.50234	-0.43168	3.594E-03	0.29
Tot				1.234E+00	98.81

E.S.I. 4. Table of volumetric source function (VSF) values for maleic dimer calculated with LBS.

atoms	X	Y	Z	VSF	VSF%
	Atomic Coordinates / (Å)				
1 O	5.610012	7.526591	0.873519	2.583E-02	5.58
2 C	6.134143	7.423601	1.996077	-2.795E-02	-6.04
3 C	6.38422	6.076718	2.577474	-2.274E-02	-4.91
4 O	6.467234	8.527969	2.64033	1.192E-02	2.58
5 H	6.044998	5.278493	1.919112	1.356E-02	2.93
6 C	6.952004	5.696463	3.739577	-1.619E-02	-3.50
7 H	6.88928	8.319174	3.561502	1.208E-03	0.26
8 H	7.020423	4.626875	3.938076	1.316E-02	2.84
9 C	7.499122	6.549814	4.818983	-1.413E-02	-3.05
10 O	7.474425	7.796555	4.825409	8.696E-03	1.88
11 O	8.016943	5.847274	5.809949	1.303E-02	2.82
12 H	8.360622	6.465131	6.545749	5.514E-03	1.19
13 O	3.823568	7.601151	4.270959	3.067E-01	66.27
14 C	4.347632	7.704149	5.393548	9.933E-03	2.15
15 O	4.680512	6.599781	6.037889	6.129E-02	13.24
16 C	4.597857	9.051021	5.974902	1.224E-02	2.64
17 H	5.102613	6.808509	6.959045	1.167E-02	2.52
18 H	4.25873	9.849336	5.31658	1.003E-02	2.17
19 C	5.165727	9.431124	7.136994	1.238E-02	2.68
20 H	5.234288	10.5007	7.335592	8.010E-03	1.73
21 C	5.712794	8.577721	8.216398	-3.967E-03	-0.86
22 O	5.687968	7.330981	8.22288	3.093E-03	0.67
23 O	6.230714	9.2803	9.207293	1.193E-02	2.58
24 H	6.574354	8.662467	9.943155	1.476E-03	0.32
Tot				4.567E-01	98.68

E.S.I. 5. Table of volumetric source function (VSF) values for acetone crystal structure calculated with PW-DFT.[a]

	Crystallographic Coordinates				
atoms	X	Y	Z	VSF	VSF%
1_O	0.300447	0.167041	0.249969	-5.335E-02	-1.91
2_O	0.699556	0.832954	0.750029	-2.534E-02	-0.91
3_O	0.300447	0.332953	0.750029	-3.069E-02	-1.10
4_O	0.699556	0.667042	0.249969	2.043E+00	73.12
5_C	0.258327	0.397683	0.249971	-5.703E-02	-2.04
6_C	0.741676	0.602312	0.750027	-1.401E-02	-0.50
7_C	0.258327	0.102311	0.750028	-1.934E-02	-0.69
8_C	0.741676	0.897684	0.24997	1.431E-01	5.12
9_C	0.224917	0.544085	0.129595	-3.377E-02	-1.21
10_C	0.775086	0.45591	0.870403	-1.474E-02	-0.53
11_C	0.224917	0.955909	0.870403	-1.540E-02	-0.55
12_C	0.775086	0.044086	0.129595	7.204E-02	2.58
13_C	0.775109	0.455911	0.62965	-1.831E-02	-0.66
14_C	0.224894	0.544084	0.370348	-3.973E-02	-1.42
15_C	0.775109	0.044083	0.370349	1.082E-01	3.87
16_C	0.224894	0.955912	0.629649	-1.842E-02	-0.66
17_H	0.292789	0.440402	0.049755	-7.179E-04	-0.03
18_H	0.707214	0.559593	0.950243	-1.820E-02	-0.65
19_H	0.292789	0.059592	0.950243	-1.277E-02	-0.46
20_H	0.707214	0.940403	0.049755	4.138E-02	1.48
21_H	0.707246	0.559596	0.54981	-2.517E-02	-0.90
22_H	0.292757	0.440399	0.450188	-4.793E-03	-0.17
23_H	0.707246	0.940398	0.450189	1.619E-01	5.79
24_H	0.292757	0.059597	0.549809	-1.612E-02	-0.58
25_H	0.050933	0.564775	0.115176	-1.316E-02	-0.47
26_H	0.94907	0.435221	0.884823	7.667E-04	0.03
27_H	0.050933	0.93522	0.884823	-6.660E-03	-0.24
28_H	0.94907	0.064775	0.115175	3.229E-02	1.16
29_H	0.949096	0.435228	0.615237	1.001E-03	0.04
30_H	0.050907	0.564767	0.384761	-1.732E-02	-0.62
31_H	0.949096	0.064767	0.384761	4.168E-02	1.49

32_H	0.050907	0.935229	0.615237	-9.550E-03	-0.34
33_H	0.291895	0.7376	0.135913	-1.512E-02	-0.54
34_H	0.708108	0.262396	0.864085	-3.939E-03	-0.14
35_H	0.291895	0.762395	0.864086	-2.380E-03	-0.09
36_H	0.708108	0.2376	0.135912	1.843E-02	0.66
37_H	0.708135	0.262394	0.635957	-6.347E-03	-0.23
38_H	0.291868	0.737601	0.364041	-1.864E-02	-0.67
39_H	0.708135	0.2376	0.364042	2.432E-02	0.87
40_H	0.291868	0.762395	0.635957	-2.596E-03	-0.09
Tot	[redacted]			2.175E+00	77.82

[a]The percentage values are calculated on the number of electrons, $n^{e}_{V_{Vdw}}$, within Van der Waals volume (V_{VdW}) equal to 2.794.

E.S.I. 6. Table of volumetric source function (VSF) values for adipic crystal structure calculated with PW-DFT.[b]

	Crystallographic Coordinates				
atoms	X	Y	Z	VSF	VSF%
1_O	0.068996	0.098631	0.219943	-9.006E-02	-0.88
2_O	0.930999	0.901372	0.780059	8.319E-01	8.11
3_O	0.893406	0.208489	0.970664	6.407E-01	6.25
4_O	0.10659	0.791513	0.029338	-1.791E-01	-1.75
5_H	0.913315	0.086287	0.904312	8.653E-03	0.08
6_H	0.08668	0.913716	0.09569	-2.839E-02	-0.28
7_C	0.975526	0.219967	0.147725	1.223E-02	0.12
8_C	0.02447	0.780036	0.852276	7.489E-03	0.07
9_C	0.93908	0.393063	0.249807	-8.264E-02	-0.81
10_C	0.060915	0.606939	0.750195	-8.489E-02	-0.83
11_H	0.767756	0.373956	0.218735	-2.263E-02	-0.22
12_H	0.232239	0.626046	0.781267	-2.723E-02	-0.27
13_H	0.997242	0.526289	0.18939	-4.432E-02	-0.43
14_H	0.002753	0.473714	0.810612	-3.070E-02	-0.30
15_C	0.035183	0.41882	0.453107	-1.246E-01	-1.22
16_C	0.964812	0.581182	0.546894	-1.398E-01	-1.36
17_H	0.207427	0.454875	0.482153	-3.018E-02	-0.29
18_H	0.792568	0.545128	0.517848	-3.328E-02	-0.32
19_H	0.989751	0.27802	0.507828	-5.764E-02	-0.56
20_H	0.010244	0.721983	0.492174	-6.671E-02	-0.65
21_O	0.463022	0.561115	0.202273	8.324E-02	0.81
22_O	0.536973	0.438887	0.797729	-4.102E-02	-0.40
23_O	0.610507	0.76022	0.021997	5.041E+00	49.16
24_O	0.389489	0.239782	0.978005	-3.155E-01	-3.08
25_H	0.582702	0.631311	0.940998	1.034E-01	1.01
26_H	0.417294	0.368692	0.059004	-8.391E-03	-0.08
27_C	0.545315	0.727968	0.170128	3.726E-02	0.36
28_C	0.45468	0.272034	0.829874	-2.536E-02	-0.25
29_C	0.58732	0.914913	0.293875	1.788E-01	1.74
30_C	0.412675	0.085089	0.706127	6.867E-03	0.07
31_H	0.543662	0.028783	0.208987	8.660E-02	0.84

32_H	0.456333	0.971219	0.791015	-6.255E-02	-0.61
33_H	0.759127	0.96194	0.349638	1.431E-01	1.40
34_H	0.240868	0.038062	0.650363	6.274E-02	0.61
35_C	0.478977	0.898876	0.445678	6.422E-01	6.26
36_C	0.521018	0.101127	0.554324	6.117E-01	5.97
37_H	0.534621	0.792904	0.534721	6.874E-02	0.67
38_H	0.465374	0.207098	0.465281	1.537E-01	1.50
39_H	0.309005	0.84023	0.389006	1.805E-01	1.76
40_H	0.690991	0.159772	0.610996	1.478E-01	1.44
Tot				7.554E+00	73.67

[b]The percentage values are calculated on the number of electrons, $n^{e}_{V_{Vdw}}$, within Van der Waals volume (V_{VdW}) equal to 10.254.

E.S.I. 7. Table of volumetric source function (VSF) values for maleic crystal structure calculated with PW-DFT.[c]

	Crystallographic Coordinates				
atoms	X	Y	Z	VSF	VSF%
1_O	0.470477	0.773054	0.718504	-1.181E-02	-0.69
2_O	0.52953	0.226944	0.281495	9.411E-01	55.27
3_O	0.529589	0.273114	0.781567	-5.157E-02	-3.03
4_O	0.470418	0.726884	0.218433	-1.101E-02	-0.65
5_O	0.891458	0.746287	0.133666	-1.407E-02	-0.83
6_O	0.108549	0.25371	0.866333	-2.896E-02	-1.70
7_O	0.108612	0.246325	0.366409	-7.488E-02	-4.40
8_O	0.891396	0.753673	0.63359	-1.280E-02	-0.75
9_O	0.626202	0.579779	0.86427	-4.431E-02	-2.60
10_O	0.373805	0.420219	0.13573	1.488E-01	8.74
11_O	0.373861	0.079835	0.635816	-1.290E-02	-0.76
12_O	0.626146	0.920163	0.364183	-2.364E-02	-1.39
13_H	0.733499	0.641041	0.973158	5.515E-04	0.03
14_H	0.266509	0.358957	0.026842	9.254E-03	0.54
15_H	0.266559	0.141094	0.52692	-1.444E-03	-0.08
16_H	0.733448	0.858904	0.473079	3.907E-04	0.02
17_O	0.1538	0.845576	0.395156	-3.279E-02	-1.93
18_O	0.846207	0.154422	0.604843	2.916E-03	0.17
19_O	0.846291	0.345613	0.104904	-2.172E-02	-1.28
20_O	0.153716	0.654384	0.895096	-2.418E-02	-1.42
21_H	0.286926	0.824873	0.531469	-2.260E-04	-0.01
22_H	0.713081	0.175125	0.46853	-2.975E-03	-0.17
23_H	0.71316	0.324918	0.968592	1.135E-03	0.07
24_H	0.286847	0.67508	0.031407	-1.268E-03	-0.07
25_C	0.136765	0.602529	0.385807	-2.432E-02	-1.43
26_C	0.863242	0.397469	0.614193	2.781E-02	1.63
27_C	0.863309	0.102568	0.114267	-8.989E-03	-0.53
28_C	0.136698	0.89743	0.885732	-5.662E-03	-0.33
29_H	0.036104	0.523384	0.288352	-9.321E-03	-0.55
30_H	0.963903	0.476614	0.711648	9.371E-03	0.55
31_H	0.963962	0.023413	0.211715	1.753E-03	0.10

32_H	0.036045	0.976584	0.788284	-3.929E-03	-0.23
33_C	0.309904	0.564826	0.557802	-1.802E-02	-1.06
34_C	0.690103	0.435172	0.442197	2.123E-01	12.47
35_C	0.690162	0.06488	0.942273	-9.157E-03	-0.54
36_C	0.309845	0.935118	0.057727	-6.525E-03	-0.38
37_H	0.335288	0.458774	0.587156	-3.205E-03	-0.19
38_H	0.66472	0.541224	0.412844	3.732E-02	2.19
39_H	0.664761	0.95883	0.912903	2.229E-03	0.13
40_H	0.335246	0.041168	0.087097	-6.556E-03	-0.39
41_C	0.473615	0.649437	0.717576	1.352E-03	0.08
42_C	0.526392	0.35056	0.282424	2.593E-01	15.23
43_C	0.52645	0.149497	0.782502	-3.379E-03	-0.20
44_C	0.473558	0.850501	0.217498	8.329E-04	0.05
45_C	0.05513	0.736075	0.299788	-7.161E-04	-0.04
46_C	0.944877	0.263922	0.700211	5.150E-03	0.30
47_C	0.944948	0.236113	0.200283	-2.777E-03	-0.16
48_C	0.055059	0.763885	0.799717	-1.741E-04	-0.01
Tot				1.188E+00	69.78

[c]The percentage values are calculated on the number of electrons, $n^{e}_{V_{vdW}}$, within Van der Waals volume (V_{VdW}) equal to 1.703.
